# Identification of D Modification Sites Using a Random Forest Model Based on Nucleotide Chemical Properties

**DOI:** 10.3390/ijms23063044

**Published:** 2022-03-11

**Authors:** Huan Zhu, Chun-Yan Ao, Yi-Jie Ding, Hong-Xia Hao, Liang Yu

**Affiliations:** 1 School of Computer Science and Technology, Xidian University, Xi’an 710071, China; hzhu0215@gmail.com (H.Z.); acy196707@163.com (C.-Y.A.); 2 Yangtze Delta Region Institute (Quzhou), University of Electronic Science and Technology of China, Quzhou 324000, China; wuxi_dyj@163.com

**Keywords:** dihydrouridine, random forest, nucleotide chemical properties, prediction, oversample

## Abstract

Dihydrouridine (D) is an abundant post-transcriptional modification present in transfer RNA from eukaryotes, bacteria, and archaea. D has contributed to treatments for cancerous diseases. Therefore, the precise detection of D modification sites can enable further understanding of its functional roles. Traditional experimental techniques to identify D are laborious and time-consuming. In addition, there are few computational tools for such analysis. In this study, we utilized eleven sequence-derived feature extraction methods and implemented five popular machine algorithms to identify an optimal model. During data preprocessing, data were partitioned for training and testing. Oversampling was also adopted to reduce the effect of the imbalance between positive and negative samples. The best-performing model was obtained through a combination of random forest and nucleotide chemical property modeling. The optimized model presented high sensitivity and specificity values of 0.9688 and 0.9706 in independent tests, respectively. Our proposed model surpassed published tools in independent tests. Furthermore, a series of validations across several aspects was conducted in order to demonstrate the robustness and reliability of our model.

## 1. Introduction

To date, over 170 RNA post-transcriptional modifications have been discovered across all domains of life [1,2,3,4,5,6,7,8,9] and play crucial roles in diverse cellular processes [10], including tRNA recognition, gene expression, metabolic responses, RNA-structure dynamics, RNA location and degradation, etc. Among them, dihydrouridine (D) is a pervasive tRNA modification that widely exists in the tRNA of eukaryotes, bacteria, and some archaea [11,12,13,14]. D has been frequently observed at conserved positions of the D-loop in tRNA [14]. The conformational flexibility of individual RNA bases can become enhanced by D [15]. The non-aromatic ring structure of the D site can result in resistance to base stacking, which may reduce the interactions with other nucleotide bases [16,17]. In addition, D has also contributed to treatments for cancerous tissues or organs [18]. Furthermore, recent work confirmed that the human tRNA-dihydrouridine synthase is related to pulmonary carcinogenesis [19]. Taken together, this evidence suggests that D plays a significant role in molecular biology and medical science.

Broadly, the precise identification of D modification sites is a fundamental process needed to conduct in-depth investigations. Researchers have used biochemical experiments to detect D modification sites since 1965 [20,21]. In recent years, high-throughput sequencing methods have become the prevailing method for detecting D modification sites [22,23]. Some popular chemical modification databases have also been established to help provide a comprehensive understanding of the potential functions of different modifications, such as RMBase (version 2.0) [7] and MODOMICS [5], which also contain information on D modifications in various species. Although biochemical methods can produce reliable and accurate information about D modification, they have typically been time-consuming and laborious [24,25,26,27]. Thus, there is an urgent need to design a high-performance computational tool for the accurate identification of D modification sites.

Until now, only three prediction tools have been available to identify RNA D modification sites. Feng et al. [28] proposed an ensemble model focused on *S. cerevisiae*. They adopted three feature extraction methods in order to encode the RNA sequence, consisting of a pseudo-dinucleotide composition (PseDNC), a nucleotide physicochemical property (NPCP), and a secondary structure component (SSC). Subsequently, the support vector machine (SVM) was used for each feature extraction method as the base model. The final ensemble model can be generated by integrating each base model with a voting score. The iRNAD investigated by Xu et al. [29] took five species into consideration. The predicting model was conducted by combining NPCP and nucleotide density features (CPND) with SVM. Recently, an original predictor called iRNAD_XGBoost was proposed by Dou et al. [30]. The hybrid sampling method, Synthetic Minority Oversampling Technique and Edited Nearest Neighbors (SMOTEEEN) [31,32], was adopted to solve the data imbalance problem. They integrated CPND, electron-ion interaction potential and pseudo-potential (EIIP and PseEIIP), Kmer, and the XGBoost-selected top 30 features in order to construct the predictor.

**Figure 1:**
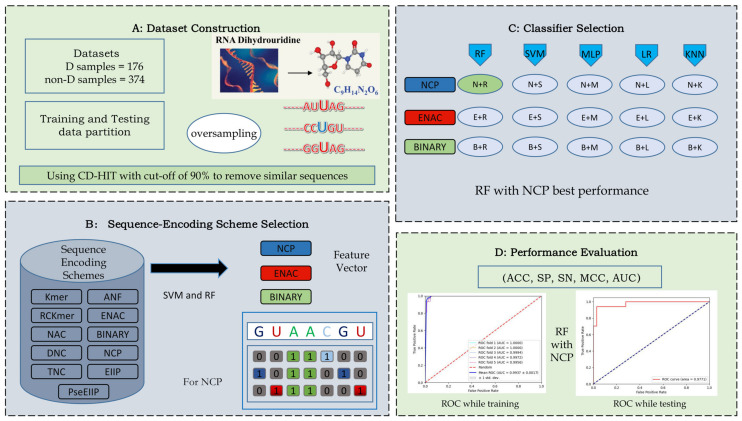
(**A**) Generation of the training and testing data partition and oversampling. (**B**) Selection of three features to encode the sequence. (**C**) Input of feature vectors into classifiers and identification the best combination of feature and classifier. (**D**) Performance evaluation with a set of metrics.

The positive and negative sample rate was imbalanced in iRNAD, which may lead to some challenges while training the model. Thus, it is necessary to obtain more balanced and reliable datasets and build more robust models. There are only two classifiers, namely SVM and XGBoost, to predict D modification sites. Considering that the scale of data is small, the deep learning algorithms are not suitable. In this study, we adopted five machine learning algorithms, including Random Forest (RF), SVM, Logistic Regression (LR), K Nearest Neighbors (KNN), and Multi-Layer Perceptron (MLP), to identify the optimal predictor. Additionally, eleven types of sequence encoding schemes were investigated, including Nucleotide Chemical Property (NCP), Enhanced Nucleic Acid Composition (ENAC), BINARY, etc. As shown in Figure 1, we first made the training and testing data partition, then oversampled the positive RNA samples with an independent sample rate of 0.5. Subsequently, we used sequence-encoding schemes to extract features and input the feature vectors into the classifiers. Ultimately, the best-performing model was identified as the combination of RF as the classifier and NCP as the encoding scheme. The fivefold cross-validation (5-fold CV) was used to train the model. Additionally, independent tests can be regarded as a means to measure the model’s generalizability.

## 2. Results

### 2.1. Sequence Encoding Scheme and Partition Rate Analysis

First, based on previous reports, we utilized two common machine learning algorithms (SVM and RF) to roughly show the performance of different sequence encoding schemes. The kernel function was chosen as RBF, while other hyper-parameters were set to the default in SVM. All machine learning algorithms underwent the 5-fold CV five times. The results of independent tests are shown in Table 1. We found that ENAC, binary, and NCP almost surpassed other methods, with a tangible improvement on all metrics in both SVM and RF. In addition, the small performance gap between these three methods indicates that these three sequence-encoding methods all captured sufficient information. Ultimately, we selected ENAC, binary, and NCP for further experiments.

It was interesting that the outcome was notably different when using different partition rates to divide the training and testing data. Here, the validation data were separated from the training data to determine hyper-parameters in the algorithm. We utilized SVM and ENAC as well as binary and NCP to choose a partition rate; 30%, 20%, and 10% were the rates chosen for the testing data.

As illustrated in Table 2, we found that almost all results improved with the decreasing of the testing partition rate in these three sequence-encoding schemes. This is probably because the amount of training data was too small to train the model and bring about under-fitting. Thus, according to this result, we selected 10% as the splitting rate to randomly separate the testing data from the raw data.

### 2.2. Oversampling and Comparison to Other Algorithms

Considering that the positive and negative samples were imbalanced, which could bias the results, there are often two ways to diminish or eliminate the impact: oversampling and down-sampling. Here, we chose oversampling because the dataset was not large enough to adopt down-sampling. We duplicated the samples of positive sequence data with an independent sample probability of 0.5 two times in the training data after partitioning. On the one hand, the duplication procedure does not change the distribution of the whole dataset. On the other hand, we expected that the model could be improved by inputting the same data multiple times with an inspiration of randomness in model training. Thereafter, we trained different classifiers with ENAC, BINARY, and NCP using the expanded data. The outcomes of the five algorithms are shown in Table 3.

It is apparent that RF performed better than other classifiers in almost all metrics. The MCC of RF reached 0.9393, and the Acc and Sp of RF-NCP were 0.9697 and 0.9706, respectively, demonstrating that the model had excellent prediction ability. Interestingly, the Sn of 0.9688 indicates that the upper bound of Sn may have been reached by several algorithms, with the exception of LR. This may be due to oversampling the positive samples. In RF, it is clear that NCP performed better than the other two encoding schemes (ENAC and BINARY) on Sn, which increased by 3.337% and 1.647%, respectively. Overall, we found that NCP outperformed the other sequence encoding schemes in multiple classifiers. Thus, we chose the combination of RF and NCP as the final model to predict D modification sites.

**Figure 2:**
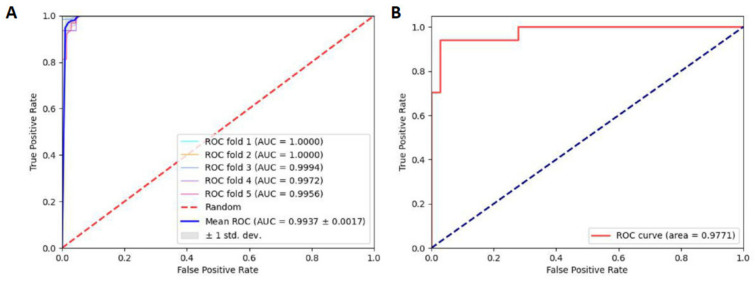
(**A**) ROC curve under the 5-fold CV. (**B**) ROC curve under independent test.

To quantitatively show the performance of the model, we utilized the AUC. The ROC curves of the combined RF and NCP model based on the 5-fold CV and independent tests are shown in Figure 2. There is a small gap between the two AUCs: the AUCs in 5-fold CV and independent tests reached 0.9937 and 0.9771, respectively, demonstrating that our model could reach satisfactory generalization ability when predicting D modification sites.

### 2.3. Robustness and Reliability Analysis

**Figure 3:**
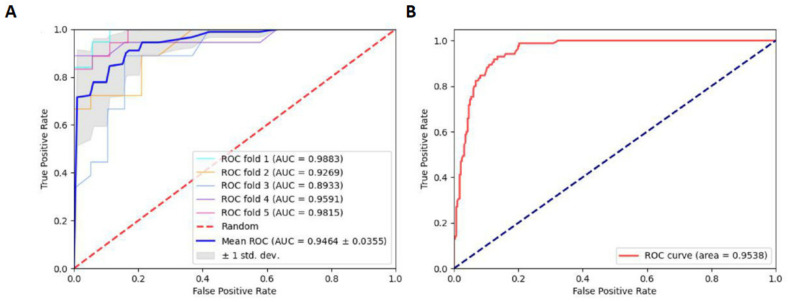
(**A**) ROC curve of the 5-fold CV of experiment I. (**B**) ROC curve of independent tests of experiment I.

Considering that approximately one-third of data originated from *S. cerevisiae*, we split the entire benchmark data into two sections: (1) training data derived only from *S. cerevisiae* and (2) the remaining four species of benchmark data, excluding *S. cerevisiae*, to serve as independent testing data. Subsequently, we trained the RF model with the NCP scheme as in experiment I. In independent tests, the values of Sn and Sp were 0.9176 and 0.8754, respectively. Furthermore, the values of MCC and Acc were 0.7249 and 0.8852, respectively. The ROC curve in experiment I was shown in Figure 3, which shows the results of the 5-fold CV and independent tests.

Relatively speaking, it is acceptable that the Acc and AUC were 0.8852 and 0.9538, respectively. This indicates that the model trained on one species can predict other species. This also suggests that it is possible to predict D sites across species. Subsequently, we also designed experiment II: the data from each species were regarded as testing data, while the remaining data were trained for the RF model.

**Figure 4:**
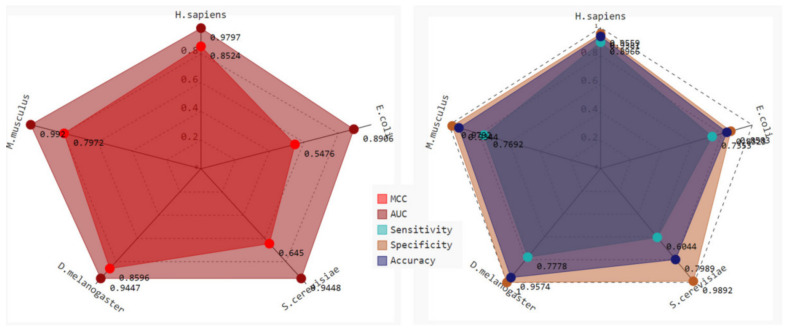
The radar map showing the performance of experiment II. The species on each corner served as the testing data, while the remaining data were used for training. Different colored radar maps indicate different metrics of performance.

As shown in Figure 4, the AUCs were considerable in all experiments, indicating that the model was sufficiently trained. However, the MCCs for *S. cerevisiae* and *E. coli* were 0.6450 and 0.5476, respectively, suggesting that the models based on these two species have poor generalizability. The AUC for *E. coli* was 0.8906, which was the lowest of all models. Taken together, this indicates that prokaryotes may possess different D modification motifs than eukaryotes.

To further survey the generalizability of our proposed model, experiment III was conducted with the goal of using each species to predict each other species. The data from each species were utilized to train a species-specific model. Particularly, for each species-specific model, the data from the other four species were individually considered as independent testing data to assess model performance.

**Figure 5:**
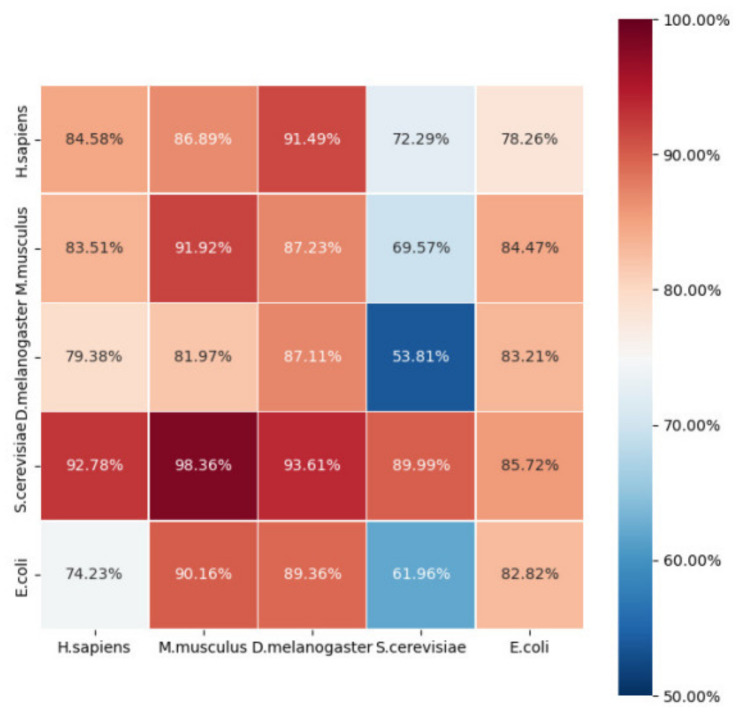
The heat map showing the species prediction accuracies (Acc values). The sample of species in the row was used to train, while the sample of species in the column served as testing.

Thus, we obtained a 55 matrix of Acc to show predictions across species. The main diagonal elements of the Acc matrix were the 5-fold CV results within species, while the remaining elements denote the prediction accuracies across species. All results are shown in Figure 5.

It is apparent that most of the prediction accuracies across species were acceptable. On the whole, the performance was relatively poor when predicting *S. cerevisiae* using models that were trained on other species. This may be because the *S. cerevisiae* dataset was larger than that of the other species. It stands to reason that a model trained on a small dataset is unable to predict large-scale data. Meanwhile, the model trained on *S. cerevisiae* performed best compared with the models trained on other species. This can also be explained by the fact that the *S. cerevisiae* dataset was larger.

The Acc was almost greater than 0.8, with the exception of *S. cerevisiae*. When predicting E. coli, the Acc was relatively poor compared with that of other species. As mentioned before, prokaryotes may have different D modification motifs from eukaryotes, which could also explain this phenomenon. To further confirm the above assumption, we searched the phylogenetic tree between the five species at http://lifemap-ncbi.univ-lyon1.fr/ (accessed on 15 December 2021). We found that *H. sapiens*, *M. musculus*, and *D. melanogaster* share a common nearest ancestor, *Bilateria*, while *S. cerevisiae* has the nearest common ancestor of *Opisthokonta*, and *E. coli* belongs to *Bacteria*. Thus, we conclude that the closer the species are phylogenetically, the better the performance of our model in cross-species prediction.

On the basis of the above result, we can conclude that the number of different species used for training is a considerable factor affecting the performance of the model. The model proposed here has a better generalization ability across species for identifying D modification sites.

### 2.4. Comparisons with Other Tools

There are three published tools to detect D modification sites. Considering that the datasets of iRNAD_XGBoost and ours came from iRNAD, Table 4 only compared our model with iRNAD and iRNAD_XGBoost in independent tests to reflect the model generalizability. Compared with iRNAD, our model performed better on most metrics. Sn improved from 86.11% to 96.88%, and Sp increased from 96.05% to 97.06%. Additionally, Acc and MCC also improved, with high percentages of 4.42% and 13.25%, respectively. The higher the MCC, the better the predictive power of a model. In addition, precision was increased from 89.19% to 96.29% compared with iRNAD_XGBoost.

## 3. Materials and Methods

### 3.1. Benchmark Datasets

It is crucial to obtain valid benchmark datasets, keeping in mind that high-quality datasets can produce incredible outcomes. In this research, we directly employed the benchmark datasets assembled by Xu et al. [29]. The datasets consist of 550 RNA samples, consisting of 176 positive RNA samples and 374 negative RNA samples. According to Xu’s research, the potential D site-containing RNA samples, derived from five species, were fetched from the RMBase (version 2.0) [7] and MODOMICS [5] databases. Xu et al. removed the sequences with over 90% sequence similarity using the CD-HIT program [33] to avoid redundancy. The distribution of datasets is illustrated in Table 5. All RNA sequences were 41 nucleotides (nt) in length, with the D modification site in the center. Previous tests indicated that the optimal prediction result for identifying D modification sites was obtained when the sequence length was set as 41 nt. The benchmark datasets above are available at http://lin-group.cn/server/iRNAD/download.php (accessed on 15 December 2021).

### 3.2. Sequence Encoding Scheme

After obtaining the data, we selected several sequence-encoding schemes to extract features. Six major types of features exist [34]. In this study, we primarily utilized eight kinds of RNA primary sequence-derived features and three nucleotide physicochemical properties to extract features, including ENAC [34,35], NCP [36], BINARY [34,35], Kmer, RCKmer [30,34], Nucleic Acid Composition (NAC), Di-Nucleotide Composition (DNC), Tri-nucleotide composition (TNC) [37,38,39,40,41], Accumulated nucleotide frequency (ANF) [29], EIIP, and PseEIIP [30]. The iLearn and iLearnplus toolkits [42] were employed to implement these encoding methods. Here, we mainly introduced NCP, BINARY, and ENAC.

#### 3.2.1. Nucleotide Chemical Property

As is well known, there are four kinds of nucleotides in RNA: adenine (A), cytosine (C), guanine (G), and uracil (U). The chemical binding and chemical structure of each nucleotide differ greatly [43]. On the basis of these chemical properties (Table 6), the four nucleotides can be tiered into three distinct groups. (1) The nucleotides can be grouped according to the ring structure; guanine and adenine are purines, which contain two rings, whereas uracil and cytosine contain only one. (2) They can be grouped in terms of the functional group; cytosine and adenine contain an amino group, whereas uracil and guanine contain a keto group. (3) They can be grouped by taking the hydrogen bond into consideration; the hydrogen bond between G and C is stronger than that between U and A.

On the basis of the above chemical properties, we could convert an RNA sequence into a discrete vector. Without loss of generality, we represented the four nucleotides (A, G, C, U) by the coordinates (1, 1, 1), (0, 1, 0), (1, 0, 0), and (0, 0, 1) respectively. Assuming that the length of the sequence was N, the dimension of the encoding vector using NCP was (N*3), and each item in the encoding vector was 0 or 1, as given below:

(1) $$R_{1}{=[r}_{1}{\;r}_{2}{\;r}_{3}\;\cdots {\;r}_{i}\;\cdots {\;r}_{N*3}{]}^{T}$$

#### 3.2.2. Binary

Binary [34,35] encoding is a familiar method that can exactly depict the position of each nucleotide in a given sample sequence. Each distinct nucleotide in an RNA sequence can be encoded into a binary vector with a length of 4 because there are four different nucleotides. Without a loss of generality, we represented the four nucleotides (A, G, C, U) by the coordinates (1, 0, 0, 0), (0, 1, 0, 0), (0, 0, 0, 1), and (0, 0, 0, 1), respectively. For instance, the RNA sequence ‘GAGACU’ can be represented by [01001000…….0001]^T^. Therefore, a 41 nt RNA sequence will be converted into a two-dimensional matrix with a size of 16 × 4.

#### 3.2.3. Enhanced Nucleic Acid Composition

Nucleotide composition (NC) [37,38,39,40,41] is a well-known set of classic encoding methods aiming to represent the preliminary features of the nucleotide sequence, and it is often adopted to count the frequency of occurrence for given K-neighboring nucleotides. As a consequence, we could obtain a dimensional feature vector for a given Kmer, which is one of the most fundamental methods used with NC. The Kmer descriptor can be calculated as follows:

(2) $$f(n_{1}n_{2}\cdots n_{k})=\frac{N(n_{1}n_{2}\cdots n_{k})}{L},(n_{k}\in (A,G,C,U))$$

where $n_{1}n_{2}\cdots n_{k}$ represents a Kmer nucleotide segment, $N(n_{1}n_{2}\cdots n_{k})$ is the count of occurrences of $n_{1}n_{2}\cdots n_{k}$ in the sequence, and L is the length of the RNA sequence.

On the basis of NC, we can derive the NAC, DNC, and TNC, while K can be chosen as 1, 2, or 3, respectively. Exactly as is the case for TNC, ENAC also corresponds to the 3-mer nucleotide frequency pattern. As a variation of NAC, ENAC integrates NAC with a sequence window, of which the window length is alterable. The entire feature vectors can be acquired by continuously window sliding from the 5′ to 3′ terminus of each nucleotide sequence. According to previous work [44], the window size is often set to 5 as a default and can be changed depending on specific prediction models as a role of hyper-parameter.

### 3.3. Classifiers

In this study, we utilized five commonly employed machine learning classifiers to screen out the optimal prediction model, including RF [45,46,47], SVM [9,45], MLP [45,48,49], KNN [50], and LR [51]. These algorithms are widely used in a range of bioinformatics research with outstanding performance.

The fundamental principle of SVM [9,45] is converting the input vectors into a high-dimensional Hilbert space, where a linear separating hyperplane can be found to separate the input into different classes. The conversion procedure can be utilized by the kernel function, which is often considered to select a radial basis kernel function (RBF).

LR [51] is a type of generalized linear model that is also used in binary classification. Based on the linear regression, LR implements a sigmoid function to convert the output of the linear regression into a value with a range of 0–1. As a result, a classification can be made with a threshold of 0.5.

MLP [45,48,49] is also known as an artificial neural network. With the exception of the input and output layer, there are often several hidden layers. Full connection is adopted among layers. The role of the activation function is implemented by the sigmoid function, aiming to separate the linearity features between layers. If there is no activation function, the whole computation can be presented by a linear vector.

KNN [50] is one of the most famous classification algorithms. As the nearest neighbors show, the decision of classification adopts a voting idea in that the category with the most neighbors is considered the final decision. It is important to choose the value of K, which is often determined by cross-validation with a lower validation error.

RF [45,46,47] integrates multiple randomly constructed independent decision trees, each of which is often regarded as a weak base learner, and holds the idea that multiple weak learners aggregated together can be comparable with strong but complex algorithms. To maintain the diversity of base learners, each base learner can be produced by randomly choosing not only the attributes but also the distribution. The introduction of attribute perturbation contributes to expanding the difference between independent decision trees. Thus, the generalization performance of the final ensemble is further improved. The tree grows as much as possible, recursively repeating the process of tree splitting until it reaches the termination condition. In the splitting period, there are two cases in which to quit splitting: (1) the size at that node is too small; (2) the execution of the splitting process is not beneficial to gain more information. The final classification of the random forest depends on the voting of multiple base learners.

### 3.4. Performance Evaluation

Cross-validation is commonly adopted to assess the performance of the constructed model while training [30,40,52]. In this study, we adopted the 5-fold CV to train the model. Additionally, an independent test was also performed to measure the generalizability of the model.

Four metrics were adopted in previous research, which have served the function of the quantitative performance evaluation of a model: (1) sensitivity (Sn); (2) specificity (Sp); (3) overall accuracy (Acc); and (4) Mathew’s correlation coefficient (MCC), as given below [53,54,55,56,57]:

(3) $$\left\{\begin{array}{ll} Sn=1-\frac{N_{-}^{+}}{N^{+}} & 0\leq Sn\leq 1\\ Sp=1-\frac{N_{+}^{-}}{N^{-}} & 0\leq Sp\leq 1\\ Acc=1-\frac{N_{-}^{+}+N_{+}^{-}}{N^{+}+N^{-}} & 0\leq Acc\leq 1\\ MCC=\frac{1-\left(\frac{N_{-}^{+}+N_{+}^{-}}{N^{+}+N^{-}}\right)}{\sqrt{\left(1+\frac{N_{+}^{-}-N_{-}^{+}}{N^{+}}\right)\left(1+\frac{N_{-}^{+}-N_{+}^{-}}{N^{-}}\right)}} & -1\leq MCC\leq 1 \end{array}\right.$$

where $N^{+}$ represents the entire number of true D site-containing sequences, while $N^{-}$ represents the entire number of the false D site-containing sequences; $N_{-}^{+}$ represents the number of D site-containing sequences that are incorrectly predicted to be false D site-containing sequences, while $N_{+}^{-}$ represents the number of false D site-containing sequences that are incorrectly predicted to be true D site-containing sequences.

In addition, the area under the curve (AUC [58]) was also adopted to quantitatively evaluate the performance of the model. The false-positive rate (1-Sp) and the true positive rate (Sn) were used to draw the receiver operating characteristic curve (ROC [58]). The larger the AUC value, the better performance the model has. Moreover, AUC = 0.5 indicates that the predictive capacity of a model is equivalent to a model using random prediction, while AUC = 1 indicates a splendid model.

## 4. Conclusions

This research screened out an effective and robust model to identify D modification sites in RNA. Oversampling and different training and testing partition rates were used to improve the performance of a model based on specific datasets. Additionally, several experiments were conducted to demonstrate the robustness and reliability of our model. Compared with iRNAD and iRNADXGBoost, for which the values of Sn were 86.11% and 91.67%, respectively, in independent tests, our model reached an Sn value of 96.88%. Moreover, corresponding MCC values had 13.25% and 9.30% improvements, while the Acc values increased by 4.43% and 3.43%, respectively. RF with NCP can be used to predict D modification sites given its satisfactory performance.

In this work, the feature extraction method was used independently instead of being integrated, which perhaps could generate more comprehensive features. There is still much to explore regarding effective feature extraction methods using integration. Inspired by the fact that simple methods are often more effective, simple duplication was performed as the traditional method of oversampling, although there may be other methods to oversample. As a considerable challenge, the issue of data imbalance always degrades model performance. It is supposed to obtain more reliable and accurate data that is balanced in both positive and negative samples. Further, deep learning algorithms are also an option to improve prediction performance when adopting large datasets. In summary, the above aspects can be further investigated to improve future research.

## Figures and Tables

**Table 1:** Performance of different sequence encoding schemes using SVM and RF in independent tests.

Performance	SVM				RF			
	Sn	Sp	Acc	MCC	Sn	Sp	Acc	MCC
Kmer	0.2955	0.8772	0.7152	0.2056	0.5161	0.6176	0.5859	0.1255
RCKmer	0.1364	0.9298	0.7089	0.1044	0.5263	0.8056	0.7091	0.3415
NAC	0.1136	**0.9912**	0.7468	0.2459	0.4063	0.7910	0.6667	0.2072
DNC	0.2955	0.8772	0.7152	0.2056	0.5806	0.7794	0.7172	0.3542
TNC	0.5682	0.8772	0.7911	0.4630	0.7368	0.9444	0.8727	0.7133
ANF	0.4773	0.8947	0.7785	0.4102	0.6316	0.8889	0.8000	0.5449
ENAC	**0.8864**	0.9737	**0.9494**	**0.8727**	0.8947	0.9722	0.9455	0.8786
BINARY	0.8636	0.9474	0.9241	0.8110	0.8065	**1.0000**	0.9394	0.8609
NCP	0.8636	0.9474	0.9241	0.8110	**0.9063**	0.9851	**0.9596**	**0.9071**
EIIP	0.6818	0.8860	0.8291	0.5718	0.8125	**1.0000**	0.9394	0.8636
PseEIIP	0.5682	0.6754	0.6456	0.2236	0.7368	0.9444	0.8727	0.7133

**Table 2:** Performance of ENAC, BINARY, and NCP with different testing data partition rates by SVM.

Performance		Sn	Sp	Acc	MCC
Encoding Scheme	Testing Data Partition Rate				
ENAC	30%	0.2623	0.9100	0.6646	0.2308
	20%	0.6111	0.8136	0.7368	0.4327
	**10%**	**0.8947**	**0.9167**	**0.9091**	**0.8021**
BINARY	30%	0.5738	0.9600	0.8137	0.6044
	20%	0.5556	0.9322	0.7895	0.5446
	**10%**	**0.7895**	**0.9722**	**0.9091**	**0.7975**
NCP	30%	0.6957	**0.9818**	0.8974	0.7482
	20%	0.7857	0.9524	0.9011	0.7632
	**10%**	**0.8421**	0.9722	**0.9273**	**0.8379**

**Table 3:** Performance of different classifiers with ENAC, BINARY, and NCP.

Performance		Sn	Sp	Acc	MCC
Algorithm	Encoding Scheme				
RF	ENAC	0.9375	0.9706	0.9545	0.9093
	BINARY	0.9531	0.9559	0.9545	0.9090
	NCP	**0.9688**	**0.9706**	**0.9697**	**0.9393**
SVM	ENAC	0.9063	0.8235	0.8333	0.6670
	BINARY	0.8438	**0.8824**	0.8939	0.7882
	NCP	**0.9688**	0.8529	**0.9091**	**0.8247**
KNN	ENAC	**0.9688**	0.8235	0.8939	0.7978
	BINARY	0.9531	0.7059	0.8258	0.6764
	NCP	**0.9688**	**0.8676**	**0.9167**	**0.8384**
LR	ENAC	0.8594	0.8235	0.8409	0.6827
	BINARY	**0.9063**	0.8382	0.8712	**0.7449**
	NCP	0.7500	**0.9552**	**0.8889**	0.7406
MLP	ENAC	0.9219	0.7941	0.8561	0.7197
	BINARY	0.9219	**0.8971**	0.9091	0.8186
	NCP	**0.9688**	**0.8971**	**0.9318**	**0.8663**

**Table 4:** Comparisons between iRNAD, iRNAD_XGBoost, and our current model to identify D modification sites in independent tests.

Tools	Sn (%)	Sp (%)	Acc (%)	MCC	AUC	Pre (%)	F1
**iRNAD**	86.11	96.05	92.86	0.83	0.98	N/A	N/A
**iRNAD_XGBoost**	91.67	94.74	93.75	0.86	0.87	89.19	**0.90**
**This work**	**96.88**	**97.06**	**96.97**	**0.94**	**0.98**	**96.29**	0.85

**Table 5:** The distribution of D in five species.

Species	*H. sapiens*	*M. musculus*	*D. melanogaster*	*S. cerevisiae*	*E. coli*
**Pos**	29	13	9	91	34
**Neg**	68	48	38	93	127

**Table 6:** Chemical properties of each nucleotide [36].

Chemical Properties	Classes	Nucleotides
Ring Structure	Pyrimidine	U, C
	Purine	G, A
Functional Group	Keto	U, G
	Amino	C, A
Hydrogen Bond	Weak	U, A
	Strong	G, C

## Data Availability

The datasets analyzed are available at http://lin-group.cn/server/iRNAD/download.php (accessed on 15 December 2021).
